# Thermodynamics of oxygen in dilute liquid silver–tellurium alloys

**DOI:** 10.1007/s00706-012-0771-z

**Published:** 2012-08-25

**Authors:** Justyna Nyk, Bogusław Onderka

**Affiliations:** Laboratory of Physical Chemistry and Electrochemistry, Faculty of Non-Ferrous Metals, AGH University of Science and Technology, 30 Mickiewicza Ave, Krakow, Poland

**Keywords:** Galvanic cell, Liquid solution, Silver–tellurium alloys, Oxygen activity, Solid electrolyte

## Abstract

**Abstract:**

The activity coefficient of oxygen $f_{\text{O}}^{0}$ in liquid Ag and binary Ag–Te dilute alloys were determined between 1,285 and 1,485 K by coulometric titration using the electrochemical cell (Ir, [O] in liquid metal or alloy | yttria stabilized zirconia | air, Pt). The experimental and evaluation procedures described in the literature were adopted. The oxygen activity coefficient was determined in pure liquid silver to be $\ln f_{\text{O}}^0  = {{ - 1{,}760.2} \mathord{\left/ {\vphantom {{ - 1,760.2} T}} \right. \kern-\nulldelimiterspace} T} + 0.6315$. Next, the oxygen activity coefficient in dilute Ag–(Te)–O alloys for variable *X*_Te_ content (from 0.01 to 0.06) was measured. From the obtained results, Wagner’s interaction parameter $\varepsilon_{\text{O}}^{\text{Te}}$ as a function of temperature was derived in the form $\varepsilon _{\text{O}}^{{\text{Te}}} \left( { \pm 0.32} \right) = {{ - 18,483} \mathord{\left/ {\vphantom {{ - 18,483} T}} \right. \kern-\nulldelimiterspace} T} + 7.668$. The electrochemical coulometric titration method seems to be very useful to study the thermodynamics of oxygen interaction in liquid silver and its alloys.

**Graphical abstract:**

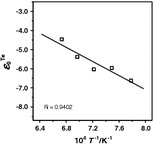

## Introduction

The by-product of the electrolytic refining of copper, i.e., “anode slime” [1], is a valuable source of silver, gold, platinum group metals, selenium, and tellurium. The processing of slime usually involves many steps with the common goal of removing Cu, Se, Te, etc., and leaving pure Ag, Au, and Pt-group metals as raw silver—so-called doré metal. So the anode slime, containing precious metals, is dried and then charged into a rotary Kaldo furnace, in which it is melted, converted, and fire-refined [2]. The product of this process is the doré metal, from which anodes are casted for elimination of remaining impurities in an electrorefining process. Pure silver cathodes contain 99.99 % Ag.

Tellurium in silver slimes is a harmful impurity element, because of limited volatilization during converter blow (contrary to selenium and bismuth), and it tends to accumulate in the metal phase. As a result of the low stability of oxides it cannot be oxidized easily during the Kaldo process in a rotary furnace.

Such conventional processing by smelting of slimes in converters with the addition of fluxes and oxidizing agents and with the injection of oxygen into the molten bath reduces the amount of Cu, Se, and Te. The refining step of the liquid alloy by oxidation is very efficient for selenium, whereas oxidation of tellurium does not take place until the Se/Te mass ratio decreases to 0.55 [3, 4]. Thus, the knowledge of oxygen interaction in Ag–Te–O liquid alloys is a key in the Ag purification process, but no previous information is available on the tellurium–oxygen interactions in molten silver. Additionally, oxygen and other dissolved gases in the liquid alloy can have a great effect on the chemical, mechanical, and electronic properties of materials. Thus the knowledge of oxygen activity in the wide composition and temperature range of liquid multi-component alloys can help one to design and optimize the conditions for the preparations of new innovative materials.

It is worth noting that as far as we know from the literature study, no measurements have been carried out to determine the interaction parameter of oxygen in liquid silver–tellurium alloys.

The modified coulometric titration method is suitable for the determination of oxygen concentration and activity in liquid metals, e.g., Ag [5] or Te [6, 7]. As silver oxide is unstable at elevated temperatures [8, 9], it seems to be the only method for the measurement of the activity of oxygen in unsaturated liquid Ag-based alloys. An additional problem is connected with the high tellurium partial pressure at elevated temperatures in the range of dilute liquid solutions of Te in silver [10].

The activity coefficient of oxygen in liquid silver has been determined several times [5, 11–25]. Some results were obtained by use of modified titration technique [5, 15, 16, 25] which is especially suitable for the determination of low oxygen concentrations and activities in liquid metallic alloys. Published solubility and activity data of oxygen in liquid silver were recently reviewed by Assal et al. [9] in their study on the thermodynamic properties of the Ag–O system.

The objective of this study was to determine experimentally the influence of Te impurity on the activity coefficient of oxygen in liquid Ag–Te alloys and to describe it by means of the Wagner [26] interaction parameter $\varepsilon_{\text{O}}^{\text{Te}}$.

Because of nonexistence of silver oxides at elevated temperatures it is impossible to quench the oxygen saturated solutions of Ag in order to analyze them. Therefore, the electrochemical coulometric titration method seems to be appropriate to study the thermodynamics of oxygen interaction in liquid silver and its alloys in a wide range of oxygen partial pressures.

## Results and discussion

In the present study the experimental method of high temperature coulometric titration with a galvanic cell was employed for the investigation of the activity coefficient of oxygen in silver-based liquid solutions. The applied oxygen concentration cell with YSZ (yttria stabilized zirconia) solid electrolyte can be described schematically as

1 $$\left( {p_{{\text{O}}_2 } } \right),{\text{Ag}}{-}\left( {{\text{Te}}} \right){-}{\text{O}}\left| {{\text{ YSZ }}} \right|{\text{O}}_{\text{2}} \left( {p'_{{\text{O}}_{\text{2}} }  = 0.213\;{\text{bar}}} \right)$$

where $p_{{{\text{O}}_{2} }}$ and $p'_{{\text{O}}_{\text{2}} }$ (in bar) are the oxygen partial pressures at the liquid metal–electrolyte interface and in the reference electrode, respectively. The electrical charge contributed by ionic current passing through the cell was recorded and analyzed.

The initial electromotive force (emf) of galvanic cell *E*_1_ (in volts) of the concentration cell is connected with the oxygen partial pressure, $p_{{{\text{O}}_{2} }}$, over liquid metal in the form

2 $$ E_{1} = \frac{R\,T}{4F}\,\,\ln \left( {\frac{0.213}{{p_{{{\text{O}}_{ 2} }} }}} \right) $$

where *R* and *T* are the gas constant and experimental temperature (in kelvin), respectively, and *F* is Faraday’s constant.

The final open circuit emf, *E*_2_, is related to the activity coefficient and final oxygen concentration by Eq. (3):

3 $$ \begin{gathered} E_{2} = \frac{RT}{2F}{ \ln }\frac{{\sqrt {0.213} }}{{f_{\text{O}} \times C_{{{\text{O}}(2)}} }} \hfill \\ \end{gathered} $$

where *C*_O(2)_ is the final oxygen concentration (in at.%) in the liquid metal or alloy and *f*_O_ is an oxygen activity coefficient in pure liquid silver rescaled for concentration in at. %. At infinite dilution the value of the oxygen activity coefficient $f_{\text{O}}^{0}$ is related to oxygen activity by Henry’s law in the following way:

4 $$ a_{\text{O}} = f_{\text{O}}^{0} \times C_{{{\text{O}}[{\text{in}}\,\,{\text{at}}.\,{\text{\% }}]}} $$

The oxygen concentration change in the sample during the titration experiment can be calculated from Eq. (5):

5 $$ C_{\text{O(1)}} - C_{\text{O(2)}} = \frac{{100 \times M \times Q_{\text{ion}} }}{2 \times W \times F} $$

where *M* and *W* are the reduced molar mass and reduced sample mass of the liquid metal or alloy, respectively. *C*_O(1)_ is an initial oxygen concentration (in at.%) in the liquid metal or alloy. The charge transferred, *Q*_ion_, caused by ionic current, *I*_ion_, was obtained by integrating the obtained curve of *I*_ion_ versus time. The ionic current passing through the cell should be corrected by subtracting the final residual current from the total cell current. It can be assumed that such constant residual current is a part of the cell current due to electron transfer in the electrolyte.

From the Nernst equation, Eq. (3), which relates oxygen concentration in the melt and the emf of the cell prior to and after titration, we can derive the following equation:

6 $$ \Updelta E = E_{2} - E_{1} = {\frac{RT} {2F}} \,  \text {ln} \left ( {\frac {{C_{\text{O(1)}} }} {{C_{\text{O(2)}}}}} \right )  $$

Equation (6) is valid under the assumption of Henry’s law. By use of Eqs. (5) and (6), the oxygen concentration in the liquid alloy can be calculated and the activity coefficient of oxygen can be determined.

The standard Gibbs free energy of oxygen dissolution in liquid metallic melt according to the reaction

7 $$ {\raise0.5ex\hbox{$\scriptstyle 1$} \kern-0.1em/\kern-0.15em \lower0.25ex\hbox{$\scriptstyle 2$}}O_{2} \;(1.01\,\,bar)\;\; \leftrightarrow \;\;\underset{\raise0.3em\hbox{$\smash{\scriptscriptstyle-}$}}{O} \;(1\;at.\,\% )\,_{in\,\,Ag} $$

can be calculated from the activity coefficient of oxygen in the form

8 $$ \Updelta G_{\text{O}}^{0} = RT\ln f_{\text{O}}^{0} $$

The reference state for dissolved oxygen is an infinitely dilute solution.

### Ag–O system

**Fig. 1:**
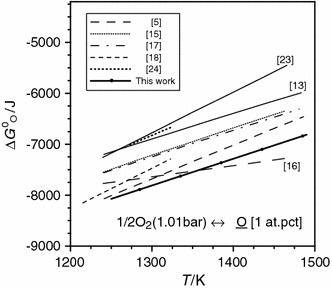
Standard Gibbs energy of solution of oxygen in liquid silver. The published experimental data were superimposed

The solubility of oxygen in Ag was measured at the beginning to test the assembly and to obtain the reference state to study the influence of tellurium on the activity coefficient of oxygen in pure liquid silver. The titration experiments were carried out twice in the temperature range 1,285–1,485 K using solid oxide galvanic cells represented schematically by Eq. (1). The standard Gibbs energies of solution of oxygen in liquid silver $\Updelta G_{\text{O}}^{0}$ for reaction (7) were calculated from these results for low values of *E*_1_. The averaged values of $\Updelta G_{\text{O}}^{0}$ obtained in the series of runs are given as a function of temperature

9 $$ \Updelta G_{\text{O}}^{0} (T) = - 14{,}635 + 5.250 \times T\,\;( \pm 370)/J $$

and are compared in Fig. 1 with the experimental data of Otsuka and Kozuka [5], Parlee and Sacris [13], Diaz et al. [15], Fischer and Ackermann [16], Shah and Parlee [17], Baker and Talukdar [18], Oberg and Friedman [23], and Otsuka and Kozuka [24].

The compared data are in reasonable agreement given the typical experimental errors of such measurement, i.e., ±500 J. Also, the temperature dependence of the free energy of dissolution seems to be in good agreement with averaged data.

The oxygen activity coefficient in pure liquid silver was determined to be

10 $$\ln f_{\text{O}}^0  = {{ - 1{,}760.2} \mathord{\left/ {\vphantom {{ - 1,760.2} T}} \right. \kern-\nulldelimiterspace} T} + 0.6315$$

### Ag–Te–O system

Next, the oxygen activity coefficients in dilute Ag–(Te)–O alloys for variable *X*_Te_ content (from 0.01 to 0.06) were measured in the temperature range 1,285–1,485 K using the following concentration galvanic cells with the solid YSZ zirconia electrolyte:

11 $$ {\text{Kanthal}} + {\text{Ir}},{\text{O in Ag}} {-} {\text{Te }}\left| {\text{ YSZ }} \right|{\text{air}},{\text{ Pt}} $$

As an example, the experimental results for 2 at.% of Te and temperature 1,385 K including the calculated ln *f*_O_ values are shown in Table 1. From these results one can observe that tellurium addition to the melt decreases the value of the activity coefficient of oxygen.

**Table 1:** Initial *E*_1_ emf value of galvanic cell of 2 at.% Te in liquid Ag–Te–O alloy at 1,385 K

Run no.	*E*_1_/V	*C*_O(1)_/at.% O	ln *f*_O_	$\Updelta G_{\text{O}}^{0}$/J
1	0.145	0.0880	−0.749	−8,623
2	0.161	0.0673	−0.750	−8,641
3	0.161	0.0673	−0.752	−8,656
4	0.152	0.0783	−0.749	−8,621
5	0.142	0.0926	−0.730	−8,405
6	0.167	0.0609	−0.749	−8,622
7	0.187	0.0436	−0.740	−8,523
For pure Ag				
8	0.117	0.1361	−0.639	−7,363

Initial oxygen concentration *C*_O(1)_, oxygen activity coefficient, average Gibbs free energy of oxygen dissolution in liquid dilute Ag-Te–O solution

To study the influence of admixture of tellurium on the activity coefficient of oxygen in dilute liquid silver alloys the dependence of oxygen on tellurium mole fraction was established in the form of Eq. (12):

12 $$ \Updelta \ln f_{\text{O}} = \ln f_{\text{O}} - \ln f_{\text{O}}^{0} = g(X_{\text{Te}} ) $$

where$\,f_{\text{O}}^{0}$ corresponds to the activity coefficient of oxygen in pure liquid silver. The obtained results, summarized in Table 2, were used to determine $\Updelta \ln f_{\text{O}}$ of oxygen as a function of composition for selected experimental temperatures.

**Table 2:** Dependence of ∆ln *f*_O_ on at.% Te and temperature *T*

at.%Te	∆ln *f*_O_				
	1,285 K	1,335 K	1,385 K	1,435 K	1,485 K
1.0	−0.1466	−0.1254	−0.1182	−0.1504	−0.1220
2.0	−0.1159	−0.0828	−0.0796	−0.0647	−0.0665
2.0	−0.1258	−0.0984	−0.1060	−0.0768	−0.0773
3.0	−0.2339	−0.1490	−0.1123	−0.1113	−0.0506
3.0	−0.2438	−0.1646	−0.1387	−0.1471	−0.0614
4.0	−0.3227	−0.3247	−0.2825	−0.2599	−0.2486
4.0	−0.3326	−0.3403	−0.3089	−0.2719	−0.2594
5.0	−0.2537	−0.2207	−0.1853	−0.2052	−0.1823
6.0	−0.2592	−0.2764	−0.3528	−0.2497	−0.1944

Diaz et al. [15] determined that oxygen in liquid silver obeys Henry’s law in experimental range of concentration. The activity coefficient can be expressed in terms of molar fraction as suggested originally by Wagner [26]; hence, the first-order interaction parameter $\varepsilon_{\text{O}}^{\text{Te}} \,$is defined by Eq. (13):

13 $$ \varepsilon_{\text{O}}^{\text{Te}} = \left( {\frac{{\partial \ln f_{\text{O}} }}{{\partial X_{\text{Te}} }}} \right)_{{X_{\text{Te}} \to 0}} $$

**Fig. 2:**
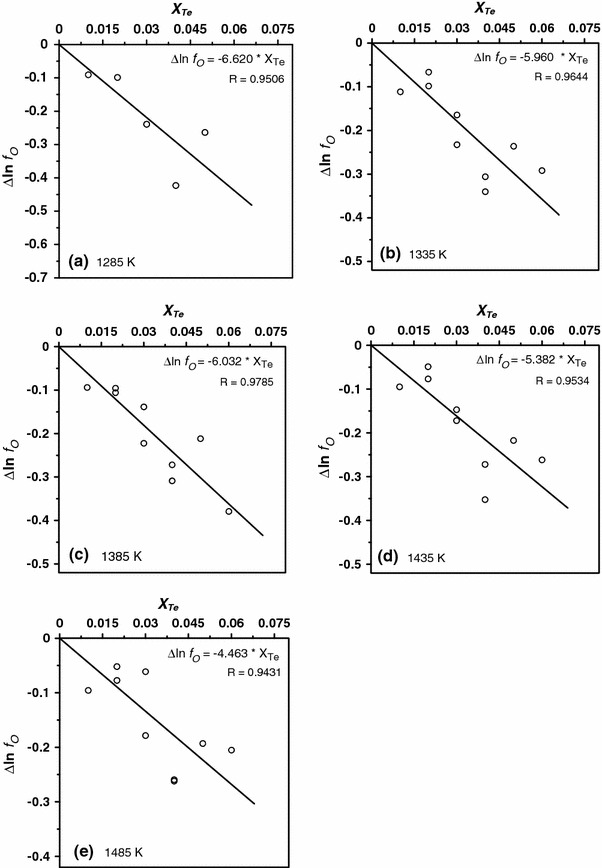
Dependence of ∆ln *f*_O_ on *X*_Te_ determined for temperatures **a** 1,285 K, **b** 1,335 K, **c** 1,385 K, **d** 1,435 K, **e** 1,485 K. Experimental data *open circles*, *solid line* linear regression, *R* coefficient of determination

For selected temperatures the parameters $\varepsilon_{\text{O}}^{\text{Te}} \,$were obtained from experimental data by the least-squares method from correlation between $\Updelta \,ln\,f_{O}$ of oxygen dissolved in liquid silver–tellurium alloys and tellurium concentration *X*_Te_ (Fig. 2).

The source of observed scatter of results in Fig. 2 is probably due to Te concentration change during the experiment in Ag dilute solutions, especially for the samples with the highest tellurium concentration. It should be noted that some of the samples were prepared for Te concentration very near the limit of a miscibility gap of liquid Ag–Te solutions. Though the evaporation of tellurium during the experiment was not observed it was possible that small changes of sample concentration took place during the heating of the galvanic cell to the desired temperature. Such titration experiment scatter was also previously observed for liquid solutions with one highly volatile element [7, 27, 28]. Additionally, the observed scatter of results could be due to the small differences in ion conductivity of different electrolyte tubes.

As a final result of this study the temperature dependence of first-order interaction parameters of oxygen in liquid silver–tellurium solutions, $\varepsilon_{\text{O}}^{\text{Te}}$, was derived in the form of an inverse temperature function

14 $$\varepsilon _{\text{O}}^{{\text{Te}}} \left( { \pm 0.32} \right) = {{ - 18,483} \mathord{\left/ {\vphantom {{ - 18,483} T}} \right. \kern-\nulldelimiterspace} T} + 7.668$$

**Fig. 3:**
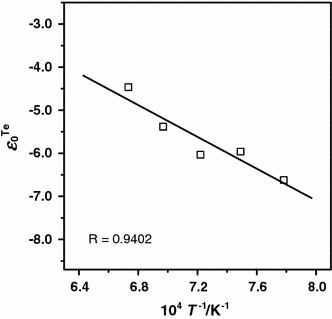
Oxygen interaction parameter $\varepsilon_{\text{O}}^{\text{Te}}$ as a function of temperature.* R* coefficient of determination

The experimental results of $\varepsilon_{\text{O}}^{\text{Te}} \,$are tabulated in Table 3 and are shown together with Eq. (14) in Fig. 3.

**Table 3:** Temperature dependence of first-order interaction parameter $\varepsilon_{\text{O}}^{\text{Te}}$

*T*/K	$\varepsilon_{\text{O}}^{\text{Te}}$
1,285	−6.620 ± 0.764
1,335	−5.960 ± 0.578
1,385	−6.032 ± 0.449
1,435	−5.382 ± 0.602
1,485	−4.463 ± 0.556

## Experimental

The scheme of an oxygen concentration cell with zirconia solid electrolyte YSZ represented by Eq. (1) can be found elsewhere [7]. The 400-mm-long one-end closed tubes of dimensions 8 mm OD (5 mm ID) of solid electrolyte ZrO_2_ (+5 mass percent Y_2_O_3_) were supplied by Friatec AG (Germany). The purity of silver rod (Alfa Aesar, Germany) used in the present investigation was 99.95 %. The iridium wire tip (diameter 0.5 mm) electrically welded to Kanthal™ wire (Kanthal AB, Sweden) was used as the lead wire for liquid silver and silver–tellurium alloys. The tellurium was added in the form of silver telluride supplied by Sigma-Aldrich (USA) but its purity was not mentioned. The metal sample was prepared from the appropriate amounts of pure silver and/or silver telluride by melting together in the electrolyte tube. The outer platinum electrode was separated into two connections (sensing and reference electrodes) in order to keep the emf measurements at the reference electrode out from polarization during a coulometric titration run. The platinum wires of outer electrodes were obtained from the Polish Mint (Poland). Before each experiment the electrolyte tube was flushed by purified argon via an H-shape tube for 10–12 h and then the furnace was slowly heated to the desired temperature.

The initial oxygen concentration in the liquid metal and alloys was set at a preselected value by passing the appropriate current through the cell. After stabilization of the open circuit voltage, *E*_1_, the steady liquid metal or alloy was deoxidized by applying a chosen voltage Δ*E* and the final emf *E*_2_ was detected. During such titration experiments the electric current and the quantity of electrical charge were controlled by an electrochemical analyzer CHI 600C Series (CH Instruments, Inc., USA) and multimeter Keithley 2001 (Keithley Instruments, Inc., USA) connected to a PC computer with acquisition software.

The quantity of electrical charge contributed by corrected *I*_ion_ was calculated by integrating the remaining current over time. Titration experiments were carried out several times at constant temperature and then the temperature was changed by a defined step and the whole procedure was repeated.

The separately measured temperature dependence of emf, $\Updelta E_{\text{corr}}$, for platinum–Kanthal junction is

15 $$\Updelta E_{\text{corr}} (V)\;\, = 2.3636 \times 10^{ - 4} + 5.458 \times 10^{ - 7} \times T - 8.32 \times 10^{ - 9} \times T^{2}.$$

This was added as a thermoelectric correction to all recorded experimental emf values of galvanic cells. As the Kanthal–Ir junction is in the constant temperature zone of the furnace, the thermo-emf due to Kanthal–Ir may be neglected.
